# The Invention of Domiciliary Benefit

**Published:** 1914-03-07

**Authors:** F. W. Francis

**Affiliations:** Chairman of the Sanatorium Sub-Committee of Warwickshire Insurance Committee


### THE EDITOR'S LETTER-BOX.

### "The Invention of Domiciliary Benefit."

*To the Editor of* The Hospital.

Sir,—From the paragraphs in your last issue, in one of which the phrase heading this letter occurs, it might easily be supposed that domiciliary benefit was invented by the London and other Insurance Committees. It is treatment at the patient's own home, and includes visits and treatment (by tuberculin, drugs, vaccines, etc., if advised) by the patient's own medical adviser—in London, I believe, under the direction of a tuberculosis expert. It also covers the accessories of treatment, such as milk, extra nourishment, cod-liver oil, drugs, dentistry, etc., and in our county, where gardens are fairly plentiful, very often a rotatory shelter with bedding, and may include nursing. In our county, in which there are something like a hundred thousand insured persons, we attach so much value to home treatment that, in addition to the forty beds we have secured in sanatoria, we are paying medical practitioners of the county something like £2,500 a year for home treatment; and for the accessories of this home treatment, judging by our decisions last week, we shall probably pay some £500 a year more. No doubt this amount will be reduced when we get dispensaries into working order. It is to be remembered domiciliary treatment allows of the provision of appliances for the surgical treatment of tuberculosis, and we are providing expensive spinal jackets in several cases. This home treatment, it is to be remembered, is in accordance with the famous Interim Report of the Parliamentary Committee on Tuberculosis, para. 18 (3), which says:

"A large proportion of cases of pulmonary tuberculosis, and some cases of other forms of tuberculosis, can be adequately treated in the patient's own home." Again, I would quote para. 28 (3): "In the case, at all events, of insured persons, patients living at home who are treated at or under the supervision of the dispensary should generally be placed, where they are willing, under the care of some general practitioner, who will carry out the necessary home treatment in consultation with the chief tuberculosis officer of the dispensary, and who will, where the patients are insured persons, be paid out of the funds available for sanatorium benefit."

It is hard on the medical practitioners of the panel, who in this county are working zealously in the treatment of tuberculosis, to be told by your valuable Journal that their services to insured persons are a mockery. The alternative is, of course, the appointment of a whole-time medical service for home treatment of tuberculosis. For this the profession at any rate is not prepared, and it is against the opinion expressed in the Report that "it is of primary importance to the lasting success of any scheme that it should enlist the hearty co-operation and stimulate the interest of the general practitioners of the country." And in Warwickshire this co-operation and interest have been secured under the present scheme.—I am, Sir, your obedient servant,

F. W. Francis,
Chairman of the Sanatorium Sub-Committee of Warwickshire Insurance Committee.

*** [We thank Mr. Francis for his letter. Mr. Francis has missed our point, which was that some Insurance Committees are condemned as incompetent and cruel through the abuse of domiciliary benefit. Some Insurance Committees in various parts of the country are highly competent and helpful; but others, like those we have condemned, are unfortunately not so. As to home treatment, that, of course must largely depend upon the nature of the case and the circumstances and surroundings of the home. It may be highly dangerous to the patient and sometimes to members of the family, or it may be adequate and justifiable in county districts such as Warwickshire, providing the conditions of each case are favourable. We favour the general use of movable shelters or chalets in villages, the results from which, within our knowledge, have been excellent. Domiciliary benefit may, however, not only be of real benefit to a patient suffering from tuberculosis, but a positive danger, as in an instance we gave; and similar cases might be multiplied. The cases which Mr. Francis seems to have chiefly in view are not of the type of those we referred to. We did not state, as Mr. Francis supposes, that all domiciliary treatment is a mockery; but we did most emphatically insist that it is a mockery to give an acute case of tuberculosis, in the first stage of that disease, so-called domiciliary benefit in the form which was imposed upon the cases which were enumerated on page 580 of last week's Hospital. It is not easy to diagnose tuberculosis in its earliest stage, and evidence increases that the sometimes hurried, often rule-of-thumb methods of selection to be met with under National Insurance, are to be condemned as unscientific and dangerous to many patients, whose lives might have been saved if they had received adequate treatment under proper conditions at the commencement of the disease. As matters often stand at present domiciliary benefit in many first-stage cases results in the gradual and sometimes rapid spread of tuberculosis with fatal results. National Insurance, where cases of tuberculosis in the first stage are concerned, as long as it fails to provide for the immediate detection and adequate treatment of all such tuberculous patients, must prove in practice a danger to the public and a cruelty to sufferers. We would further point out that Mr. Francis is hardly entitled to claim special credit for spending £2,500 a year on medical advice for home-treated patients; for he admits having 100,000 insured in his area, and £2,500 is exactly equal to 100,000 sixpences, the capitation fee for domiciliary treatment which the doctors are allowed by law.—Ed., The Hospital.]

